# Discovering Associations: Kawasaki Disease and COVID-19

**DOI:** 10.1155/2020/8880242

**Published:** 2020-09-28

**Authors:** Nicholas Peterson, Kaustubh Sagdeo, Donna Tyungu, Cristin Harper, Kyle Mihaylo, Elza Pollak-Christian

**Affiliations:** ^1^The University of Oklahoma Health Sciences Center, Department of Pediatrics, Section of Pediatric Hospital Medicine, Oklahoma City, OK 73104, USA; ^2^The University of Oklahoma Health Sciences Center, Department of Pediatrics, Pediatric Resident, Oklahoma City, OK 73104, USA

## Abstract

The COVID-19 pandemic has resulted in over 3.6 million confirmed cases and over 254,000 deaths worldwide. It has been theorized that children who are asymptomatic or who do not display significant respiratory symptoms are potential vectors for community transmission of the SARS-CoV-2 virus. This is incompletely understood due to the current lack of widespread testing in the pediatric population. We describe a case of a 2-year-old female who presented with symptoms of prolonged fever, conjunctivitis, extremity edema, rash, dry/cracked lips, fussiness and fatigue, and a notable absence of respiratory symptoms. She was diagnosed with and treated for Kawasaki disease. Due to her prolonged fever, she was tested for COVID-19 which was positive; however, she did not develop respiratory symptoms during her illness. At the time of manuscript submission, this is the second case report to our knowledge showing an association between Kawasaki Disease and SARS-CoV-2 virus, both of which are poorly understood diseases in the pediatric population. This case highlights the value of testing pediatric patients for COVID-19 who present with fever in the absence of other symptoms to improve epidemiologic measures during the ongoing pandemic, and it also adds to a foundation of cases for future research on the presence of a link between Kawasaki Disease and COVID-19.

## 1. Introduction

The coronavirus disease of 2019 (COVID-19) pandemic has resulted in over 3.6 million confirmed cases and over 254,000 deaths [1]. Studies have shown that pediatric patients represent a small percentage of confirmed cases [2]. At the time of the writing of this manuscript, it is theorized that the pediatric population may play a role in community transmission of the SARS-CoV-2 virus [3]. We present a case showing an association between Kawasaki disease and the SARS-CoV-2 virus. This case highlights the value of testing patients for COVID-19 during evaluation for Kawasaki disease (KD).

## 2. Case Description

A previously healthy and fully immunized 2-year-old female presented to the emergency room for prolonged fever, conjunctival erythema, hand swelling, rash, dry/cracked lips, fussiness, and fatigue. At symptom onset, she developed a low-grade fever and fatigue. On days 4-5 of fever, she developed mild bilateral hand swelling, bilateral nonexudative conjunctivitis, dry/cracked lips, and a scant, maculopapular rash on her distal upper and lower extremities. On day nine, she developed the classic “strawberry tongue” papillitis. She neither developed any significant upper or lower respiratory symptoms nor any noticeable lymphadenopathy. Throughout her illness, she had no known ill or febrile contacts, although she continued to attend daycare until three days before fever onset. The patient's father had just completed a two-week self-isolation period after returning home from South Korea. He had not exhibited fever or any other symptoms of illness. The patient's mother had been working in a respiratory clinic during the COVID-19 pandemic, where patients presented for COVID-19 testing if they exhibited concerning symptoms; she reported wearing proper personal protective equipment (PPE) during all patient encounters at her workplace. Her last shift occurred two days prior to the patient's admission.

On day ten of fever, the patient presented to a local emergency department. Her laboratory testing showed a normal white blood cell count (WBC), normocytic anemia (hemoglobin 9.4 mg/dL), thrombocytosis (498,000 cells/mm^3^), elevated inflammatory markers (ESR 58 mm/hr, CRP 89.9 mg/L), hypoalbuminemia (2.9 g/dL), elevated ALT (64 U/L), and mild pyuria on urinalysis (5–10 WBC/hpf). Rapid group A streptococcal testing was negative, and a one-view chest radiograph showed no significant abnormalities. She was subsequently transferred to our tertiary children's hospital for direct admission for suspected KD. Before the patient's arrival, the decision was made by the pediatric hospitalist and pediatric infectious disease attending to send COVID-19 PCR testing on arrival. On admission, she was afebrile and with normal vital signs for age, and examination was notable for mild bilateral conjunctivitis, dry lips, mildly swollen hands and feet, and a healing scant papular rash on all extremities.

Kawasaki disease was diagnosed when the patient met four out of five clinical criteria (bilateral nonexudative conjunctivitis, erythematous oral mucosal changes, peripheral extremity swelling, and generalized rash noted on extremities) in the setting of her prolonged fever. An echocardiogram was normal without evidence of coronary artery dilatation or decreased cardiac function. She was given a single dose of 2 g/kg intravenous immunoglobulin (IVIG) and started on medium-dose acetylsalicylic acid (ASA, 44 mg/kg/day) per current recommendations [4].

COVID-19 PCR test result was positive within ten hours of the patient's admission. Infection prevention and control and the transfer hospital were immediately notified of the result. The patient's mother was instructed to notify all of the patient's known contacts over the past two weeks so that they could self-isolate and monitor for symptoms. The patient did not develop any respiratory symptoms during admission and was discharged after a 24-hour resolution of fever. She was transitioned to low-dose ASA (∼5.5 mg/kg daily, for ease of dosing) with plans to follow-up with pediatric cardiology for repeat echocardiogram two weeks after discharge. Her family was instructed to quarantine at home for fourteen days from her positive test date. Written informed consent to publish the patient's case was obtained from her parents prior to discharge.

## 3. Discussion

Human coronaviruses (HCoVs) are ubiquitous and are thought to cause the “common cold” in children with mild symptoms of the upper respiratory tract [5]. HCoVs are also capable of acute severe respiratory illness outbreaks predominantly affecting the adult population, such as the severe acute respiratory syndrome coronavirus (SARS-CoV), the Middle East respiratory syndrome coronavirus (MERS-CoV), and the current COVID-19 pandemic caused by the SARS-CoV-2 virus. Similar to the prior SARS and MERS outbreaks, SARS-CoV-2 virus causes fewer and less severe symptoms in otherwise healthy children. Pediatric cases are associated with a much lower mortality rate than adults [2, 3, 6]. Children have the same likelihood as adults to become infected with COVID-19, but the majority of pediatric cases are asymptomatic or show a relatively mild course with a good prognosis [2, 6].

There is prior published evidence such as winter-spring prevalence in regions with nontemperate climates that suggest that KD may be triggered by respiratory viral infections [7]. Prior large cohort studies have found that up to 42% of children with KD have a positive respiratory viral PCR [5, 7, 8]. These studies also found that HCoVs are associated with up to 7.1% of KD cases [5]. This is comparable to studies that also showed a 7% positive rate for HCoVs in a random cohort of asymptomatic children [8]. Hence, the evidence for HCoV as a causative trigger for KD is inconclusive.

The first published case of KD and COVID-19 infection reported a 6-month-old child with five days of fever and typical clinical findings meeting criteria for classic KD [9]. Due to fever, nasal congestion, and a chest radiograph finding of a faint opacity in the left lung, the patient was tested for COVID-19. She was treated with IVIG and ASA and discharged home. Her COVID-19 test resulted positive after she was diagnosed and treated for KD. In our case, apart from having a prolonged fever, the patient did not display any other symptoms typical for COVID-19. COVID-19 testing resulted early in the course of admission but did not alter the management of the patient. Her positive COVID-19 status did allow for increased vigilance in infection control measures during her admission and contact tracing in order to reduce the spread of COVID-19.

This patient's presentation emphasizes the need for more widespread testing. It is notable that this patient would not have screened for COVID-19 testing according to our hospital's initial testing guidelines, and may otherwise have never been tested for COVID-19 given her lack of respiratory symptoms, and the need to test selectively. Given that COVID-19 remains a disease for which there is currently no vaccine or proven cure, widespread testing and aggressive contact tracing are likely to remain paramount in controlling future outbreaks.

## 4. Conclusion

We report a case showing a link between Kawasaki disease and the SARS-CoV-2 virus. This case highlights the value of testing patients for COVID-19 during evaluation for possible Kawasaki disease. At the time of this manuscript submission, it is unclear whether or not COVID-19 triggers or alters Kawasaki disease but we have shown that discovery can provide knowledge to improve infection control practices. As clinical practice becomes more targeted regarding whom to test, healthcare providers should be aware of this association.

## Data Availability

No data were used in the manuscript.

## Abbreviations

mg: milligrams
G: grams
dL: geciliter
mm: millimeter
U: units
L: liter
hpf: high-power field
ESR: erythrocyte sedimentation rate
CRP: C-reactive protein
ALT: alanine aminotransferase.
